# Analysis of Long Noncoding RNAs-Related Regulatory Mechanisms in Duchenne Muscular Dystrophy Using a Disease-Related lncRNA-mRNA Pathway Network

**DOI:** 10.1155/2022/8548804

**Published:** 2022-12-14

**Authors:** Bing Xu, Chunlei Zheng

**Affiliations:** ^1^Department of Pediatrics, Tongxiang First People's Hospital, Tongxiang, Zhejiang 314500, China; ^2^Department of Orthopedics, Tongxiang First People's Hospital, Tongxiang, Zhejiang 314500, China

## Abstract

**Objective:**

This study aimed to investigate the molecular regulatory mechanisms underpinning Duchenne muscular dystrophy (DMD).

**Methods:**

Using microarray data, differentially expressed long noncoding RNAs (DELs) and DMD-related differentially expressed mRNAs (DEMs) were screened based on the comparative toxicogenomics database, using a cutoff of |log_2_ fold change| > 1 and false discovery rate (FDR) < 0.05. Then, protein-protein interaction (PPI), coexpression network of lncRNA-mRNA, and DMD-related lncRNA-mRNA pathway networks were constructed, and functional analyses of the genes in the network were performed. Finally, the proportions of immune cells infiltrating the muscle tissues in DMD were analyzed, and the correlation between the immune cells and expression of the DELs/DEMs was studied.

**Results:**

A total of 46 DELs and 313 DMD-related DEMs were identified. The PPI network revealed *STAT1*, *VEGFA*, and *CCL2* to be the top three hub genes. The DMD-related lncRNA-mRNA pathway network comprising two pathways, nine DELs, and nine DMD-related DEMs showed that *PYCARD*, *RIPK2*, and *CASP1* were significantly enriched in the NOD-like receptor signaling pathway, whereas *MAP2K2*, *LUM*, *RPS6*, *PDCD4*, *TWIST1*, and *HIF1A* were significantly enriched with proteoglycans in cancers. The nine DELs in this network were DBET, MBNL1-AS1, MIR29B2CHG, CCDC18-AS1, FAM111A-DT, GAS5, LINC01290, ATP2B1-AS1, and PSMB8-AS1.

**Conclusion:**

The nine DMD-related DEMs and DELs identified in this study may play important roles in the occurrence and progression of DMD through the two pathways of the NOD-like receptor signaling pathway and proteoglycans in cancers.

## 1. Introduction

Duchenne muscular dystrophy (DMD), a severe and progressive X-linked disease that is characterized by muscular dystrophy, is caused by mutations in the dystrophin gene and has a global prevalence of approximately 0.005% of male births [1]. Deficiency of the dystrophin protein in muscles results in the progressive loss of muscle tissue and impaired muscle function, rendering the muscles more vulnerable to mechanical damage [2]. Complications, including skeletal muscle wasting, cardiomyopathy, and respiratory insufficiency, are often observed, with cardiopulmonary failure being the most common cause of DMD-related mortality [3]. Multidisciplinary symptomatic treatment is used in the clinical management of DMD [4]. Gene-based therapies and myogenic cell transplantation are the current therapeutic approaches for DMD [5].

Long noncoding RNAs (lncRNAs) are noncoding transcripts longer than 200 nucleotides that are not translated into proteins [6]. LncRNAs are important modulators of messenger RNA (mRNA) and modulate mRNA expression at the posttranscriptional level by functioning as competing endogenous RNAs (ceRNAs) that competitively bind to microRNAs (miRNAs) [7]. LncRNAs regulate dystrophin expression and thus represent a potential therapeutic strategy to alleviate dystrophin protein deficiency [8]. LncRNA H19 reportedly ameliorates muscular dystrophy by suppressing the degradation of the dystrophin protein [9]. In a recent study, bioinformatics analyses were used to comprehensively examine DMD-related differentially expressed lncRNAs (DELs) by constructing a ceRNA network of lncRNA-miRNA-mRNA using gene expression omnibus (GEO) datasets [10]. However, DMD-related lncRNAs and their regulatory molecular mechanisms involving lncRNAs, mRNAs, and pathways have not been fully investigated.

In the present study, gene expression profiles of DMD samples were analyzed using a series of bioinformatics tools to identify differentially expressed mRNAs (DEMs) and DELs in DMD. Next, protein-protein interaction (PPI) networks of the DMD-related DEMs and DMD-related lncRNA-mRNA pathway were constructed. Additionally, alterations in different types of immune cells and the correlation between immune cells and crucial factors in DMD were also analyzed. Finally, the expression of the important factors (lncRNAs and mRNAs) identified in the study was verified in the training and validation sets. Thus, the study identified several potential biomarkers and therapeutic targets for DMD and has significant implications for the development of novel treatment strategies based on them.

## 2. Materials and Methods

### 2.1. Data Source and Preprocessing

The gene expression profiles from the GSE38417 and GSE6011 datasets were downloaded from the NCBI Gene Expression Omnibus (GEO, https://www.ncbi.nlm.nih.gov/geo/). The GSE38417 dataset, which was used as a training set, included muscle tissue samples from 16 patients with DMD and six normal control individuals (Affymetrix Human Genome U133 Plus 2.0 Array platform). However, the GSE6011 dataset, which served as a validation set, contained 36 muscle tissue samples (22 patients with DMD and 14 normal control individuals) and was analyzed using the GPL96 detection platform. Then, the mRNA and lncRNA expression was reannotated according to the annotation files from the Ensembl genome browser database (https://asia.ensembl.org/index.html).

### 2.2. Screening DEMs and DELs in DMD

Using the limma package of the *R* software (version 3.6.1, https://bioconductor.org/packages/release/bioc/html/limma.html), the raw expression datasets were subjected to log_2_ transformation and then normalized using the “between array normalization” function. Subsequently, DEMs and DELs were screened with a strict cutoff of false discovery rate (FDR) < 0.05 and |log_2_ fold change (FC)| > 1. Then, bidirectional hierarchical clustering analysis based on the centered Pearson correlation was performed on the identified DEMs and DELs using the pheatmap package (https://cran.r-project.org/web/packages/pheatmap/index.html) in *R* 3.6.1.

Using the keyword “Duchenne muscular dystrophy,” DMD-related genes were searched in the comparative toxicogenomics database (CTD) [11] (https://ctdbase.org/), with the inference score threshold set at >3. Following comparison with the identified DEMs, the overlapping DMD-related DEMs were selected for gene ontology (GO) terms [12] and Kyoto Encyclopedia of Genes and Genomes (KEGG) pathway [13] enrichment analyses using the online tool Database for Annotation, Visualization, and Integrated Discovery (DAVID, version 6.8, https://david.ncifcrf.gov/). An FDR of <0.05 was set as the threshold for a significant difference.

### 2.3. Constructing a PPI Network

The overlapping DMD-related DEMs identified using the screening were subjected to searches for interactions of the proteins they encoded using the search tool for the retrieval of interacting genes [14] (STRING, version 11.0, https://string-db.org/), and the PPI pairs with a combined score >0.6 were retained to construct a PPI network [15]. Cytoscape (version 3.9.0) was used to visualize the PPI network. Subsequently, the topological features of the network, including the average shortest path length, degree centrality (DC), closeness centrality (CC), and betweenness centrality (BC) [15], were calculated to determine the hub genes in the PPI network using the CentiScaPe plugin (version 2.2) of Cytoscape software (https://apps.cytoscape.org/apps/centiscape). Degree refers to the number of interactions (edges) at a node (protein), and node genes with the highest degrees were defined as hub genes.

The molecular complex detection (MCODE) version 1.4.2; https://apps.cytoscape.org/apps/mcode) plugin in the Cytoscape software was used to mine the functionally related and highly interconnected modules using the following parameters: degree cutoff = 2, node score cutoff = 0.2, and K-core = 2. Then, the functional analysis of the genes in these modules was performed using the BINGO plugin (version 2.44; https://apps.cytoscape.org/apps/bingo) in the Cytoscape software with FDR <0.05.

### 2.4. Constructing a Coexpression Network of lncRNA-mRNA

Correlations between the DELs and overlapping DMD-related DEMs were analyzed by calculating Pearson's correlation coefficient (PCC) using the cor.test (https://stat.ethz.ch/R-manual/R-devel/library/stats/html/cor.test.html) using the *R* software. Then, the lncRNA-mRNA pairs with *P* < 0.05 and PCC > 0.9 were chosen to construct a lncRNA-mRNA coexpression network, and the network was visualized using Cytoscape software. Next, the genes in the coexpression network were subjected to GO term and KEGG pathway enrichment analyses using the DAVID online tool (version 6.8) with FDR < 0.05.

### 2.5. Constructing a DMD-Related lncRNA-mRNA Pathway Network

The DMD-related KEGG pathways in the CTD database were searched using the keyword “duchenne muscular dystrophy.” The DMD-related KEGG pathways thus identified were compared with the KEGG pathways that were significantly enriched by the genes in the coexpression network, and the overlapping KEGG pathways and overlapping KEGG pathway-related DEMs and DELs were retained to construct a DMD-related lncRNA mRNA pathway network.

### 2.6. Correlation between the Crucial Factors and DMD-Related Immune Cell Types

The infiltration of immune cells in muscle tissues is an important feature of DMD [16]. The proportion of different types of immune cells infiltrating the muscle tissue of patients with DMD and normal control individuals was calculated using CIBERSORT [17] (https://cibersort.stanford.edu/index.php), and the differences between the two sets of samples were compared using *t*-tests. Finally, the correlation between the significantly different immune cell types and the expression of the factors (mRNAs and lncRNAs) in the DMD-related lncRNA mRNA pathway network was analyzed by calculating the PCC.

## 3. Results

### 3.1. Identification and Functional Analysis of DELs and DEMs in DMD

**Figure 1:**
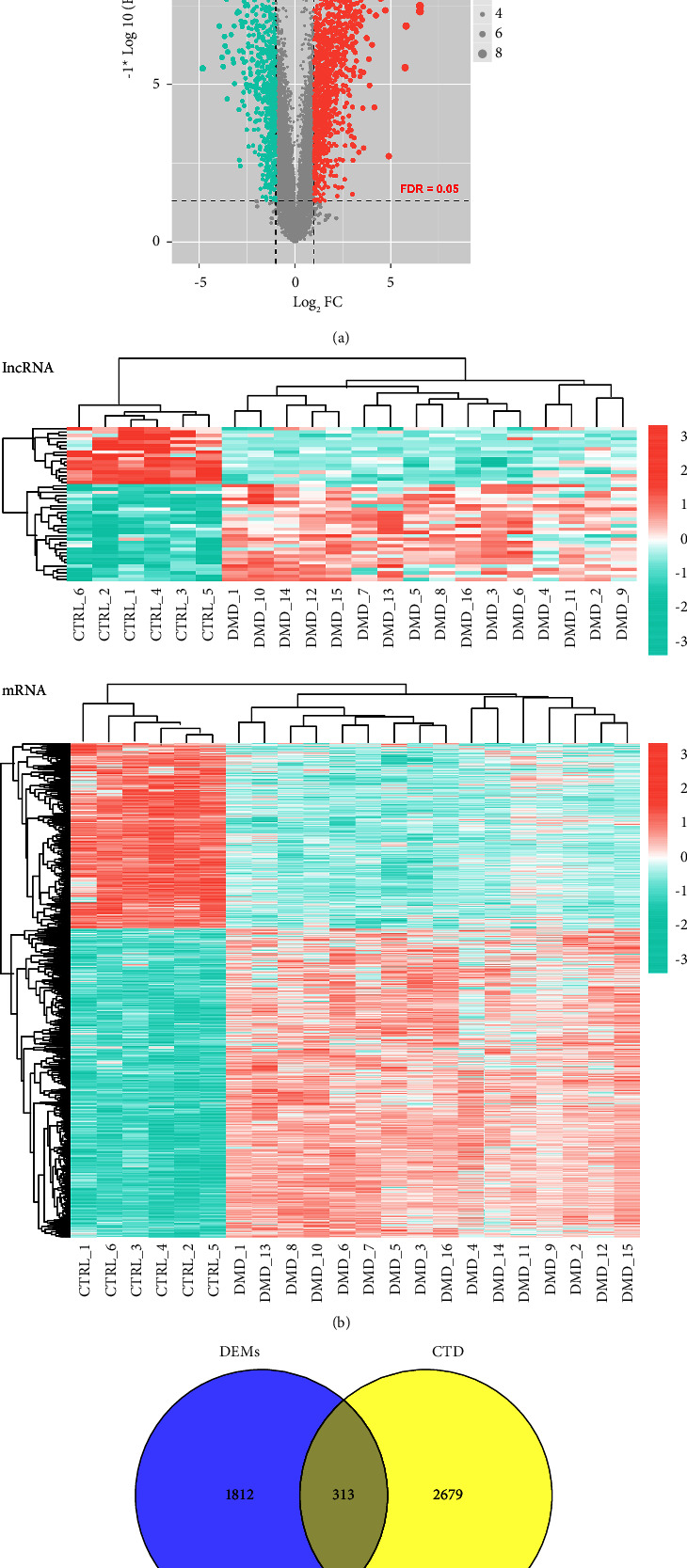
Identification of differentially expressed lncRNA (DELs) and differentially expressed mRNAs (DEMs) in Duchenne muscular dystrophy (DMD). (a) Volcano plots of DELs and DEMs in DMD based on the thresholds of false discovery rate (FDR) < 0.05 and |log_2_ fold change| > 1. Blue and red spots represent downregulated and upregulated DELs/DEMs, respectively. (b) Bidirectional hierarchical clustering heatmap of DELs (upper) and DEMs (lower). (c) A Venn diagram of DEMs and DMD-related genes identified in the comparative toxicogenomics database (CTD) with the inference score >3 set as the threshold.

Following analysis, 1708 lncRNAs and 17326 mRNAs were annotated in the GSE38417 dataset. Based on the cutoffs of FDR < 0.05 and |log_2_ FC| > 1, 46 DELs and 2125 DEMs were screened between DMD and normal control samples (Figure 1(a)). The 46 DELs and 2125 DEMs were bidirectionally hierarchically clustered, and the results showed that these DELs and DEMs effectively differentiate DMD samples from normal control samples (Figure 1(b)). Based on the CTD database, we obtained 2992 DMD-related genes with an inference score >3. These genes were compared with the 2125 DEMs, and 313 overlapping DMD-related DEMs were identified (Figure 1(c)).

**Figure 2:**
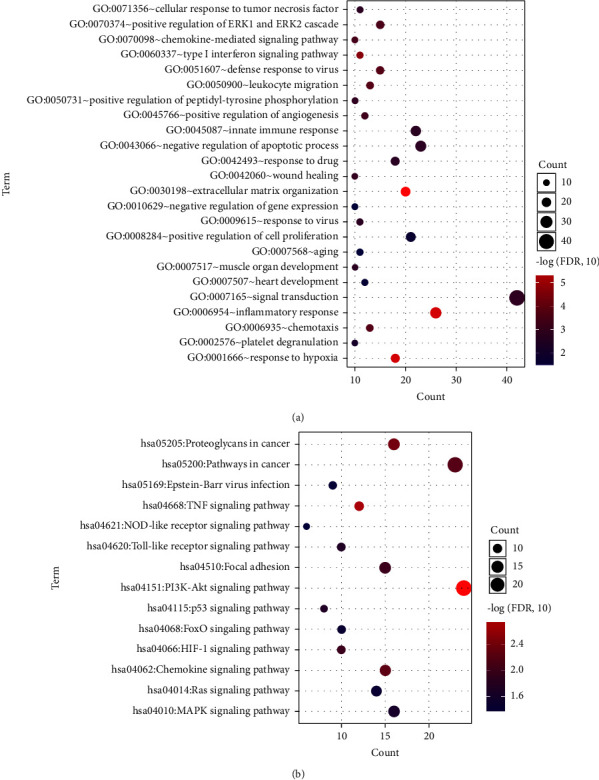
Functional analyses of the overlapping DMD-related DEMs. (a) Significantly enriched gene ontology (GO) terms of biological processes. (b) Significantly enriched Kyoto Encyclopedia of Genes and Genomes (KEGG) signaling pathways of the DMD-related DEMs. The horizontal axis indicates the number of the DMD-related DEMs, and the vertical axis indicates the name of items. The larger the dots, the greater the number of DEMs. The darker the dot, the more significant the enrichment of process.

These overlapping DMD-related DEMs were then subjected to functional analysis and found to be significantly enriched in 24 GO terms of biological processes and 14 KEGG signaling pathways (FDR < 0.05), including “positive regulation of ERK1 and ERK2,” “inflammatory response,” “response to hypoxia,” “TNF signaling pathway,” “NOD-like receptor signaling pathway,” “PI3K-Akt signaling pathway,” “p53 signaling pathway,” “FoxO signaling pathway,” “Ras signaling pathway,” and “MAPK signaling pathway” (Figures 2(a) and 2(b)).

### 3.2. Analysis of the PPI Network Based on the DMD-Related DEMs

**Figure 3:**
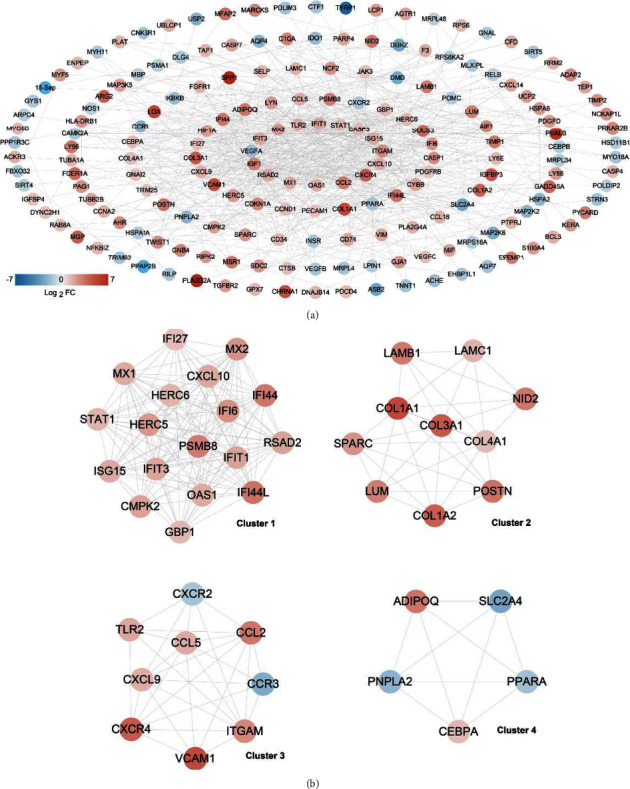
Construction of a protein-protein interaction (PPI) network based on the overlapping DMD-related DEMs. (a) A PPI network based on the DMD-related DEMs. A change in the color of the spots from blue to red indicates a change in the degree of significant difference from downregulation to upregulation. (b) Four network clusters extracted from the PPI network. Blue and red spots represent downregulated and upregulated proteins, respectively.

The 313 DMD-related DEMs were subjected to a search for interactions between the proteins encoded by these DMD-related DEMs using the STRING database, and 695 interaction pairs with a combined score >0.6 were identified and used to build a PPI network, which contained 216 nodes and 695 edges (Figure 3(a)). After calculating the centrality parameters of each node in the network, the top 20 genes were identified (listed in Table 1), and the top three hub genes were *STAT1* (degree = 41), *VEGFA* (degree = 32), and *CCL2* (degree = 28).

Furthermore, we extracted four network clusters using MCODE (Figure 3(b)), including clusters 1 (18 nodes and 133 edges), 2 (10 nodes and 33 edges), 3 (9 nodes and 29 edges), and 4 (5 nodes and 10 edges). Following enrichment and annotation of the biological processes, the significantly related biological functions of each cluster were obtained (Supplementary Table 1). Clusters 1, 2, 3, and 4 were significantly related to immune response, skeletal system development, defense response, and response to organic substances, respectively.

### 3.3. Analysis of the Coexpression Network of lncRNA mRNA

**Figure 4:**
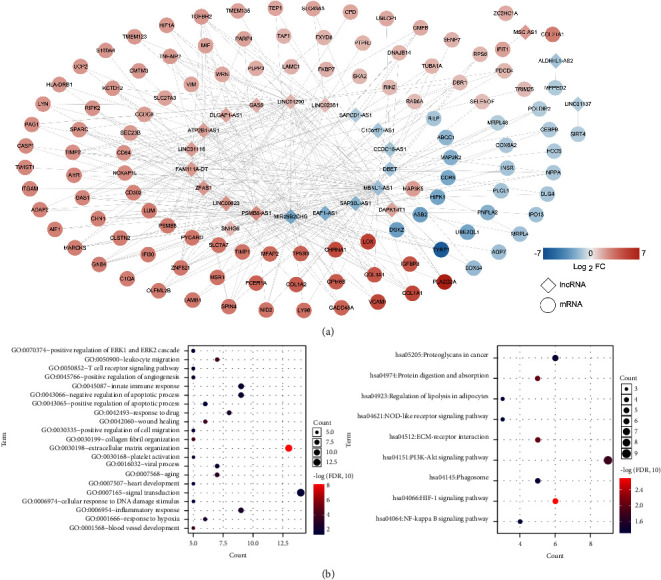
Establishment of a coexpression network of lncRNA-mRNA and functional analyses of the factors in this coexpression network. (a) A coexpression network of lncRNA-mRNA was constructed with the lncRNA-mRNA pairs (*P* < 0.05 and PCC > 0.9). Diamonds and circles represent lncRNAs and mRNAs, respectively. A change in spot color from blue to red indicates a change in the degree of significant difference from downregulation to upregulation. (b) Significantly enriched GO terms of biological process (left) and KEGG signaling pathways (right) of the genes in this coexpression network.

To investigate the relationship between the identified DELs and overlapping DMD-related DEMs in the pathogenesis of DMD, we calculated the PPCs of the DELs and overlapping DMD-related DEMs. Based on the thresholds of *P* < 0.05 and PPC > 0.9, 308 lncRNA-mRNA pairs were obtained, and a coexpression network of lncRNA-mRNA was constructed (Figure 4(a)). Next, the genes in this coexpression network were subjected to functional analysis. With the FDR set at <0.05, 21 GO terms of biological process and nine KEGG signaling pathways were found to be significantly enriched and included “positive regulation of ERK1 and ERK2,” “T cell receptor signaling pathway,” “inflammatory response,” “response to hypoxia,” “NOD-like receptor signaling pathway,” “PI3K-Akt signaling pathway,” “HIF-1 signaling pathway,” and “NF-kappa B signaling pathway” (Figure 4(b)).

### 3.4. Analysis of the DMD-Related lncRNA-mRNA Pathway Network Composed of Nine mRNAs, Nine lncRNAs, and Two Pathways

**Figure 5:**
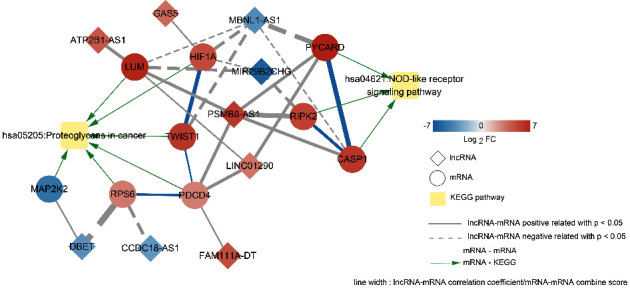
Construction of a DMD-related lncRNA-mRNA pathway network composed of two KEGG pathways, nine DMD-related DEMs, and nine DELs. Diamonds, circles, and yellow squares represent DELs, DMD-related DEMs, and KEGG pathways, respectively.

We searched the CTD database using the keyword “Duchenne muscular dystrophy” and identified 45 KEGG signaling pathways to be closely associated with DMD. Following comparison with KEGG pathways significantly enriched by the genes in the coexpression network, two overlapping KEGG pathways were obtained, including the NOD-like receptor signaling pathway and proteoglycans in cancers. Subsequently, the interaction between DEMs in the overlapping KEGG signaling pathways and the coexpression relationship with lncRNAs were integrated, and a DMD-related lncRNA-mRNA pathway network was proposed with two KEGG pathways, nine DMD-related DEMs (*LUM*, *HIF1A*, *PYCARD*, *RIPK2*, *CASP1*, *PDCD4*, *RPS6*, *MAP2K2*, and *TWIST1*), and nine DELs (ATP2B1-AS1, GAS5, MBNL1-AS1, MIR29B2CHG, PSMB8-AS1, FAM111A-DT, CCDC18-AS1, DBET, and LINC01290) (Figure 5). From the lncRNA-mRNA pathway network, *PYCARD*, *RIPK2*, and *CASP1* were found to be significantly enriched in the NOD-like receptor signaling pathway, whereas *MAP2K2*, *LUM*, *RPS6*, *PDCD4*, *TWIST1*, and *HIF1A* were significantly enriched in proteoglycans in cancers.

### 3.5. Analysis of the Proportion of DMD-Related  Immune Cell Types and Correlation between Immune Cell Types and Factors in the lncRNA-mRNA Pathway Network

**Figure 6:**
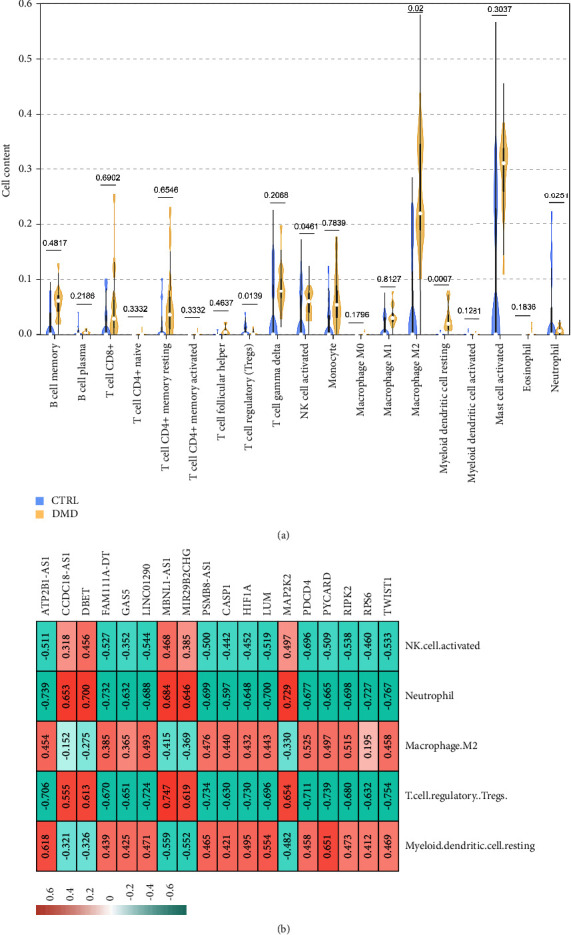
Correlation between immune cell types and the factors in the lncRNA-mRNA pathway network. (a) Proportion of the various immune cell types in the DMD samples and normal control subjects. (b) Correlation analysis between the five significantly different immune cell types (in patients with DMD and control individuals) and the factors (DELs and DMD-related DEMs) in the lncRNA-mRNA pathway network.

Based on the expression of all the genes detected in the samples, the CIBERSORT algorithm was used to calculate the proportion of various immune cell types in each sample, and 19 immune cell types were identified. The proportions of regulatory T cells (Tregs, *P*=0.0139), activated natural killer (NK) cells (*P*=0.0461), and neutrophils (*P*=0.0251) were significantly decreased, whereas the proportions of M2 macrophages (*P*=0.02) and resting myeloid dendritic cells (*P*=0.0007) were significantly increased (Figure 6(a)) in the DMD samples compared to those in the normal control samples. Furthermore, analysis of the correlation between the five significantly different immune cell types, nine DMD-related DEMs, and nine DELs in the DMD-related lncRNA-mRNA pathway network revealed that activated NK cells, neutrophils, and Tregs were positively correlated with the expression of four DELs (*CCDC18-AS1*, *DBET*, *MBNL1-AS1*, and *MIR29B2CHG*) and one DMD-related DEM (*MAP2K2*), whereas the cells were negatively correlated with the expression of the other five DELs and eight DMD-related DEMs. However, resting myeloid dendritic cells and M2 macrophages showed an opposite trend (Figure 6(b)).

### 3.6. Validation of the Identified Crucial Factors in the GSE38417 and GSE6011 Datasets

**Figure 7:**
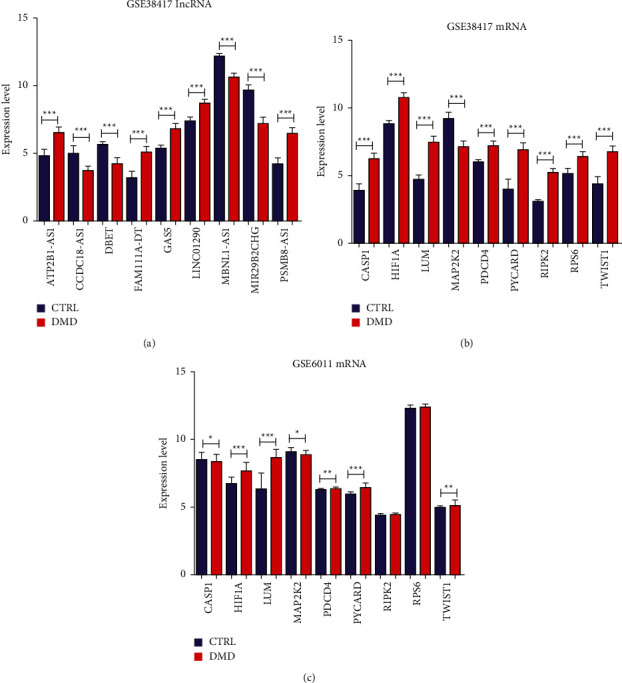
Validation of the crucial factors (lncRNAs and mRNAs) in the training (GSE38417) and validation (GSE6011) sets. (a) Expression of the nine DELs in the DMD-related lncRNA-mRNA pathway network between DMD and normal control samples in GSE38417. (b) Expression of the nine DMD-related DEMs between DMD and normal control samples in GSE38417. (c) Expression of the nine DMD-related DEMs between DMD and normal control samples in GSE6011. ^*∗*^*P* < 0.05; ^*∗∗*^*P* < 0.01; ^*∗∗∗*^*P* < 0.001.

The expression of the crucial factors in the lncRNA-mRNA pathway network was analyzed in the training (GSE38417) and validation (GSE6011) sets. In GSE38417, the expression of lncRNA ATP2B1-AS1, FAM111A-DT, GAS5, LINC01290, and PSMB8-AS1 significantly increased (*P* < 0.05), whereas that of CCDC18-AS1, DBET, MBNL1-AS1, and MIR29B2CHG was significantly decreased (*P* < 0.05, Figure 7(a)) in the DMD samples compared to that in the normal control samples. Additionally, in GSE38417, the levels of mRNA *CASP1*, *HIF1A*, *LUM*, *PDCD4*, *PYCARD*, *RIPK2*, *RPS6*, and *TWIST1* were significantly upregulated in DMD samples compared to those in normal controls (*P* < 0.05), whereas mRNA *MAP2K2* was markedly downregulated in DMD samples (*P* < 0.05, Figure 7(b)). Because GSE6011 is based on the GPL96 annotation platform, few lncRNAs could be annotated on this platform. Therefore, we only verified the expression of nine DMD-related DEMs in the validation set (GSE6011). We found that the trend of expression of these DMD-related DEMs (except for *CASP1*, *RIPK2*, and *RPS6*) in GSE6011 was consistent with that in GSE38417 (Figure 7(c)).

## 4. Discussion

DMD is a fatal X-linked genetic disorder characterized by progressive muscular wasting resulting from dystrophin protein deficiency [18]. The involvement of lncRNAs in the pathophysiology of DMD is being increasingly investigated because of their pivotal roles in the regulation of dystrophin protein expression. In the present study, we identified 46 DELs and 313 DMD-related DEMs in DMD. A PPI network was constructed based on these DMD-related DEMs, and *STAT1*, *VEGFA*, and *CCL2* were identified as the top three hub genes. Based on the DMD-related DEMs and DELs, a coexpression network of lncRNAs and mRNAs was generated, and the factors in this coexpression network were found to be significantly enriched in 21 GO terms of biological processes and nine KEGG signaling pathways. According to the CTD database, NOD-like receptor signaling and proteoglycans in cancer pathways were found to be closely related to DMD. The DMD-related lncRNA-mRNA pathway network was found to consist of two pathways, nine DMD-related DEMs and nine DELs. Furthermore, five immune cell types were found to be significantly different between the DMD and normal control samples. Thus, this study provides important insights into the underlying mechanisms of lncRNAs in the pathogenesis of DMD and provides potential targets that could aid in the rational design of lncRNA-based therapies for DMD.

Lack of dystrophin results in chronic inflammation, which is closely associated with muscle degeneration in the pathogenesis of DMD [19, 20]. In our proposed DMD-related lncRNA-mRNA pathway networks, *PYCARD*, *RIPK2*, and *CASP1* were significantly enriched in the NOD-like receptor signaling pathway, whereas *MAP2K2*, *LUM*, *RPS6*, *PDCD4*, *TWIST1*, and *HIF1A* were significantly enriched in proteoglycans in cancers. The NOD-like receptor (NLR) assembles a protein complex called the NLR family pyrin domain-containing 3 (NLRP3) inflammasome in response to certain infectious and sterile stimuli [21]. NLPR3 inflammasomes are upregulated as a result of dystrophin deficiency and play a key pathogenic role in DMD [22]. The NLR signaling pathway has a critical role in inflammation [23]. *PYCARD* encodes the inflammasome adaptor apoptosis-associated speck-like protein containing a C-terminal caspase recruitment domain (ASC), a component of the NLRP3 inflammasomes, which is involved in downstream signaling of the inflammasome pathway [24]. Receptor-interacting serine/threonine protein kinase 2 (*RIPK2*) regulates NLRP3 and inflammation [25], and the NOD/RIPK2 inflammatory signaling pathway has been found to confer susceptibility to osteoarthritis [26]. *CASP1*, encoding caspase 1, is a key component of NLRP3 inflammasomes [27] and is positively related to active ulcerative colitis [28]. Our results suggest that *PYCARD*, *RIPK2*, and *CASP1* may be involved in regulating DMD-related inflammation via the NLR signaling pathway.

The dystrophin protein is a component of the dystrophin-associated glycoprotein complex that connects actin filaments, intermediate filaments, and microtubules to transmembrane protein complexes to stabilize muscle cells [29]. Proteoglycans, a class of highly glycosylated proteins that are expressed in almost all tissues, are involved in tissue homeostasis and remodeling of the stromal microenvironment during physiological and pathological processes, such as tissue regeneration, angiogenesis, and cancer [30]. *MAP2K2* encodes mitogen-activated protein kinase 2, an important component of the MAPK pathway. The MAPK pathway is an important regulator of myofiber death [31]. MAPK/ERK signaling plays a critical role in various biological activities by phosphorylating various substrates in the cytoplasm and nucleus and has been reported to play a central role in organ regeneration [32]. Ribosomal protein S6 (*RPS6*) controls mRNA translation, and phospho-RPS6 has been identified as a surrogate marker of the activated PI3K/AKT/mTORC1 pathway found in many cancer types [33]. Programmed cell death 4 (*PDCD4*) mediates inflammation and regulates the MAPK pathway [34]. Wang et al. [35] demonstrated that the PDCD4/HO-1 pathway is involved in oxidative stress and inflammation in atherosclerosis. Lumican (*LUM*), a member of the small leucine-rich proteoglycan family, is present in muscle tissues and plays dual roles as an oncogene and tumor suppressor [36]. *TWIST1* has been reported to regulate inflammatory genes in skeletal muscle [37] and mediate epithelial-to-mesenchymal transition and cell migration [38]. The activation of HIF1*α* (encoded by *HIF1A*) is an important cause of vascular dysfunction and impaired angiogenesis in DMD [39]. Additionally, interaction between *HIF1A* and *TWIST* was observed in the DMD-related lncRNA-mRNA pathway network. *HIF1α* silencing reportedly leads to downregulation of *TWIST1* [40]. Taken together, these results suggest that *MAP2K2*, *LUM*, *RPS6*, *PDCD4*, *TWIST1*, and *HIF1A* may play important roles in DMD pathophysiology by regulating the MAPK pathway and proteoglycans in cancer.

In addition, nine DELs, including DBET, MBNL1-AS1, MIR29B2CHG, CCDC18-AS1, FAM111A-DT, GAS5, LINC01290, ATP2B1-AS1, and PSMB8-AS1, were identified in the DMD-related lncRNA-mRNA pathway network. The lncRNA DBET is associated with the epigenetic etiology of facioscapulohumeral muscular dystrophy [41]. Li et al. showed that the downregulation of lncRNA MBNL1-AS1 suppressed skeletal muscle cell apoptosis [42]. GAS5 reportedly promotes wound healing by activating the HIF1A/VEGF pathway [43]. Dysregulation of the lncRNAs MBNL1-AS1, CCDC18-AS1, LINC01290, MIR29B2CHG, and PSMB8-AS1 has been observed in many cancers [44–48]. NF-*κ*B signaling is activated during DMD pathogenesis and leads to inflammation and muscle degeneration [49]. A substantial body of evidence suggests that lncRNA ATP2B1-AS1 protects against inflammation by targeting the NF-*κ*B signaling pathway [50, 51], which implies that lncRNA ATP2B1-AS1 may ameliorate inflammation and muscle degeneration in the pathogenesis of DMD via NF-*κ*B signaling. However, the role of FAM111A-DT has not been identified. Therefore, these nine DELs may play important roles in the occurrence and development of DMD through the NLR signaling pathway and proteoglycans in cancers. However, the specific mechanisms of these nine lncRNAs in DMD pathogenesis need to be investigated further.

In DMD, the immune system is activated and several types of immune cells, such as CD4+ and CD8+ T cells, Tregs, and NK cells, invade the skeletal muscles [16]. A recent study showed that lncRNA *CCDC18-AS1* is positively correlated with activated myeloid dendritic cells and neutrophils [52], in concordance with our results that showed that the expression of lncRNA *CCDC18-AS1* was positively correlated with the proportion of activated NK cells, neutrophils, and Tregs but negatively correlated with resting myeloid dendritic cells and M2 macrophages. Our study also showed that the proportions of Tregs, activated NK cells, neutrophils, M2 macrophages, and resting myeloid dendritic cells were significantly different between patients with DMD and normal control individuals.

However, this study has some limitations. First, the number of samples in the GSE38417 dataset was small, and additional validation cohorts need to be analyzed in future studies. Second, as this study involved secondary analysis of microarray data, further experimental studies are needed to verify the results of our study. Additionally, the specific mechanisms of the identified DMD-related DEMs and DELs need to be further explored in vitro and in vivo.

## 5. Conclusions

By performing a series of bioinformatics analyses on microarray data, a DMD-related lncRNA-mRNA pathway network was constructed. Nine DELs (DBET, MBNL1-AS1, MIR29B2CHG, CCDC18-AS1, FAM111A-DT, GAS5, LINC01290, ATP2B1-AS1, and PSMB8-AS1), nine DMD-related DEMs (*PYCARD*, *RIPK2*, *CASP1*, *MAP2K2*, *LUM*, *RPS6*, *PDCD4*, *TWIST1*, and *HIF1A*), and two KEGG pathways (NLR signaling pathway and proteoglycans in cancer) were identified to play a crucial role in the pathogenesis of DMD. Our findings provide novel insights into the regulatory relationships between lncRNAs, mRNAs, and pathways in the pathogenesis of DMD. The DMD-related DEMs and DELs identified in this study need to be further investigated for their clinical use as potential biomarkers and therapeutic targets for DMD.

## Figures and Tables

**Table 1:** The centrality parameters of the top20 genes in the protein-protein interaction network.

ID	Average shortest pathlength	Betweenness centrality	Closeness centrality	Degree
STAT1	1.17	0.80	0.86	41
VEGFA	1.00	0.22	1.00	32
CCL2	2.06	0.62	0.49	28
CXCL10	2.29	0.44	0.44	27
CXCR4	2.05	1.29	0.49	27
ITGAM	1.67	0.56	0.60	26
CASP3	2.30	0.22	0.44	25
IGF1	1.67	1.02	0.60	24
MX1	1.58	0.05	0.63	20
ISG15	1.50	0.03	0.67	19
RSAD2	1.86	0.02	0.54	19
MX2	1.64	0.02	0.61	18
TLR2	0.00	0.00	0.00	18
IFIT3	1.53	0.03	0.65	18
IFIT1	1.50	0.02	0.67	18
GBP1	1.50	0.29	0.67	17
IFI44L	1.56	0.02	0.64	17
IFI44	1.53	0.02	0.66	17
CXCL9	2.48	0.46	0.40	17
PDGFRB	1.54	0.47	0.65	17

## Data Availability

The datasets used and analyzed during the current study are available from the corresponding author upon request.

## Supplementary Materials

Supplementary Materials Supplementary table 1: Detailed information of the four PPI network clusters.
