# Supplementary material for: Analysis of Long Noncoding RNAs-Related Regulatory Mechanisms in Duchenne Muscular Dystrophy Using a Disease-Related lncRNA-mRNA Pathway Network
Source: Genet Res (Camb). 2022 Dec 14;2022:8548804. doi: 10.1155/2022/8548804 (PMC9771664; doi:10.1155/2022/8548804)
Supplement: Supplementary Materials — Supplementary table 1: Detailed information of the four PPI network clusters. [file 8548804.f1.docx]

**Supplementary table 1** Detailed information of the four PPI network clusters.

| Cluster 1 |  |  |  |  |  |
| --- | --- | --- | --- | --- | --- |
| GO-ID | p-value | corr p-value | x | Description | Genes in test set |
| 9615 | 1.46E-10 | 4.45E-08 | 7 | response to virus | HERC5\|RSAD2\|STAT1\|MX2\|MX1\|IFI44\|ISG15 |
| 51707 | 2.78E-09 | 4.22E-07 | 8 | response to other organism | HERC5\|CXCL10\|RSAD2\|STAT1\|MX2\|MX1\|IFI44\|ISG15 |
| 9607 | 1.62E-08 | 1.64E-06 | 8 | response to biotic stimulus | HERC5\|CXCL10\|RSAD2\|STAT1\|MX2\|MX1\|IFI44\|ISG15 |
| 51704 | 7.04E-08 | 5.35E-06 | 9 | multi-organism process | HERC5\|CXCL10\|RSAD2\|STAT1\|MX2\|MX1\|IFI44\|ISG15\|PSMB8 |
| 6955 | 5.26E-05 | 3.20E-03 | 6 | immune response | HERC5\|CXCL10\|RSAD2\|OAS1\|IFI6\|IFI44L |
| 43281 | 1.64E-04 | 8.31E-03 | 3 | regulation of caspase activity | IFI27\|STAT1\|IFI6 |
| 52548 | 1.98E-04 | 8.59E-03 | 3 | regulation of endopeptidase activity | IFI27\|STAT1\|IFI6 |
| 52547 | 2.29E-04 | 8.70E-03 | 3 | regulation of peptidase activity | IFI27\|STAT1\|IFI6 |
| 2376 | 5.47E-04 | 1.83E-02 | 6 | immune system process | HERC5\|CXCL10\|RSAD2\|OAS1\|IFI6\|IFI44L |
| 6952 | 6.03E-04 | 1.83E-02 | 5 | defense response | HERC5\|CXCL10\|RSAD2\|MX2\|MX1 |
| 50896 | 7.17E-04 | 1.98E-02 | 11 | response to stimulus | HERC5\|CXCL10\|RSAD2\|OAS1\|STAT1\|MX2\|MX1\|IFI6\|IFI44\|ISG15\|IFI44L |
| 6919 | 2.19E-03 | 4.81E-02 | 2 | activation of caspase activity | IFI27\|STAT1 |
| 9612 | 2.26E-03 | 4.81E-02 | 2 | response to mechanical stimulus | CXCL10\|STAT1 |
|  |  |  |  |  |  |
| Cluster 2 |  |  |  |  |  |
| GO-ID | p-value | corr p-value | x | Description | Genes in test set |
| 30198 | 2.47E-11 | 5.43E-09 | 6 | extracellular matrix organization | COL1A1\|COL3A1\|POSTN\|COL1A2\|LUM\|LAMC1 |
| 43062 | 3.61E-10 | 3.97E-08 | 6 | extracellular structure organization | COL1A1\|COL3A1\|POSTN\|COL1A2\|LUM\|LAMC1 |
| 1501 | 1.50E-06 | 5.49E-05 | 5 | skeletal system development | COL1A1\|COL3A1\|POSTN\|COL1A2\|SPARC |
| 9888 | 3.57E-06 | 1.07E-04 | 6 | tissue development | COL1A1\|COL3A1\|POSTN\|COL1A2\|COL4A1\|LAMC1 |
| 48731 | 2.17E-05 | 3.75E-04 | 8 | system development | COL1A1\|COL3A1\|POSTN\|COL1A2\|SPARC\|COL4A1\|LAMB1\|LAMC1 |
| 48513 | 4.07E-05 | 5.96E-04 | 7 | organ development | COL1A1\|COL3A1\|POSTN\|COL1A2\|COL4A1\|LAMB1\|LAMC1 |
| 48856 | 4.40E-05 | 6.05E-04 | 8 | anatomical structure development | COL1A1\|COL3A1\|POSTN\|COL1A2\|SPARC\|COL4A1\|LAMB1\|LAMC1 |
| 7155 | 6.11E-05 | 7.52E-04 | 5 | cell adhesion | COL3A1\|POSTN\|LAMB1\|LAMC1\|NID2 |
| 22610 | 6.16E-05 | 7.52E-04 | 5 | biological adhesion | COL3A1\|POSTN\|LAMB1\|LAMC1\|NID2 |
| 7275 | 1.03E-04 | 1.19E-03 | 8 | multicellular organismal development | COL1A1\|COL3A1\|POSTN\|COL1A2\|SPARC\|COL4A1\|LAMB1\|LAMC1 |
| 32501 | 1.69E-04 | 1.77E-03 | 9 | multicellular organismal process | COL1A1\|COL3A1\|POSTN\|COL1A2\|SPARC\|LUM\|COL4A1\|LAMB1\|LAMC1 |
| 32502 | 1.95E-04 | 1.87E-03 | 8 | developmental process | COL1A1\|COL3A1\|POSTN\|COL1A2\|SPARC\|COL4A1\|LAMB1\|LAMC1 |
| 16043 | 4.25E-04 | 3.22E-03 | 7 | cellular component organization | COL1A1\|COL3A1\|POSTN\|COL1A2\|LUM\|LAMB1\|LAMC1 |
| 9653 | 7.77E-04 | 5.51E-03 | 5 | anatomical structure morphogenesis | COL1A1\|COL1A2\|COL4A1\|LAMB1\|LAMC1 |
|  |  |  |  |  |  |
| Cluster 3 |  |  |  |  |  |
| GO-ID | p-value | corr p-value | x | Description | Genes in test set |
| 9605 | 4.01E-11 | 1.82E-08 | 8 | response to external stimulus | CXCL9\|ITGAM\|VCAM1\|CCL5\|CXCR2\|CCL2\|CCR3\|TLR2 |
| 6952 | 1.03E-10 | 1.82E-08 | 8 | defense response | CXCL9\|VCAM1\|CCL5\|CXCR2\|CCL2\|CXCR4\|CCR3\|TLR2 |
| 6954 | 8.14E-11 | 1.82E-08 | 7 | inflammatory response | CXCL9\|VCAM1\|CCL5\|CXCR2\|CCL2\|CXCR4\|CCR3 |
| 40011 | 8.48E-10 | 6.52E-08 | 7 | locomotion | CXCL9\|ITGAM\|VCAM1\|CCL5\|CXCR2\|CCL2\|CCR3 |
| 42221 | 1.21E-09 | 8.15E-08 | 9 | response to chemical stimulus | CXCL9\|ITGAM\|VCAM1\|CCL5\|CXCR2\|CCL2\|CXCR4\|CCR3\|TLR2 |
| 9611 | 3.59E-09 | 1.97E-07 | 7 | response to wounding | CXCL9\|VCAM1\|CCL5\|CXCR2\|CCL2\|CXCR4\|CCR3 |
| 2376 | 1.76E-07 | 5.91E-06 | 7 | immune system process | CXCL9\|ITGAM\|VCAM1\|CCL5\|CXCR2\|CCL2\|TLR2 |
| 6950 | 4.40E-07 | 1.31E-05 | 8 | response to stress | CXCL9\|VCAM1\|CCL5\|CXCR2\|CCL2\|CXCR4\|CCR3\|TLR2 |
| 7166 | 1.38E-06 | 3.39E-05 | 7 | cell surface receptor linked signaling pathway | CXCL9\|ITGAM\|CXCR2\|CCL2\|CXCR4\|CCR3\|TLR2 |
| 50896 | 4.36E-06 | 7.91E-05 | 9 | response to stimulus | CXCL9\|ITGAM\|VCAM1\|CCL5\|CXCR2\|CCL2\|CXCR4\|CCR3\|TLR2 |
| 7165 | 1.88E-05 | 2.41E-04 | 7 | signal transduction | CXCL9\|CCL5\|CXCR2\|CCL2\|CXCR4\|CCR3\|TLR2 |
| 23052 | 3.79E-05 | 4.34E-04 | 8 | signaling | CXCL9\|ITGAM\|CCL5\|CXCR2\|CCL2\|CXCR4\|CCR3\|TLR2 |
| 23033 | 3.99E-05 | 4.47E-04 | 7 | signaling pathway | CXCL9\|ITGAM\|CXCR2\|CCL2\|CXCR4\|CCR3\|TLR2 |
| 23046 | 4.77E-05 | 4.94E-04 | 7 | signaling process | CXCL9\|CCL5\|CXCR2\|CCL2\|CXCR4\|CCR3\|TLR2 |
| 23060 | 4.77E-05 | 4.94E-04 | 7 | signal transmission | CXCL9\|CCL5\|CXCR2\|CCL2\|CXCR4\|CCR3\|TLR2 |
| 50794 | 7.07E-03 | 1.78E-02 | 8 | regulation of cellular process | CXCL9\|VCAM1\|CCL5\|CXCR2\|CCL2\|CXCR4\|CCR3\|TLR2 |
| 50789 | 1.03E-02 | 2.40E-02 | 8 | regulation of biological process | CXCL9\|VCAM1\|CCL5\|CXCR2\|CCL2\|CXCR4\|CCR3\|TLR2 |
| 65007 | 1.57E-02 | 3.16E-02 | 8 | biological regulation | CXCL9\|VCAM1\|CCL5\|CXCR2\|CCL2\|CXCR4\|CCR3\|TLR2 |
| 9987 | 2.22E-02 | 4.07E-02 | 9 | cellular process | CXCL9\|ITGAM\|VCAM1\|CCL5\|CXCR2\|CCL2\|CXCR4\|CCR3\|TLR2 |
|  |  |  |  |  |  |
| Cluster 4 |  |  |  |  |  |
| GO-ID | p-value | corr p-value | x | Description | Genes in test set |
| 10033 | 6.44E-05 | 1.40E-03 | 4 | response to organic substance | CEBPA\|ADIPOQ\|SLC2A4\|PPARA |
| 31325 | 9.78E-05 | 2.02E-03 | 4 | positive regulation of cellular metabolic process | CEBPA\|ADIPOQ\|PPARA\|PNPLA2 |
| 9893 | 1.20E-04 | 2.23E-03 | 4 | positive regulation of metabolic process | CEBPA\|ADIPOQ\|PPARA\|PNPLA2 |
| 42221 | 5.03E-04 | 5.08E-03 | 4 | response to chemical stimulus | CEBPA\|ADIPOQ\|SLC2A4\|PPARA |
| 48522 | 1.71E-03 | 9.46E-03 | 4 | positive regulation of cellular process | CEBPA\|ADIPOQ\|PPARA\|PNPLA2 |
| 48519 | 1.76E-03 | 9.46E-03 | 4 | negative regulation of biological process | CEBPA\|ADIPOQ\|PPARA\|PNPLA2 |
| 48518 | 2.48E-03 | 1.11E-02 | 4 | positive regulation of biological process | CEBPA\|ADIPOQ\|PPARA\|PNPLA2 |
| 44238 | 6.89E-03 | 1.98E-02 | 5 | primary metabolic process | CEBPA\|ADIPOQ\|SLC2A4\|PPARA\|PNPLA2 |
| 32502 | 1.07E-02 | 2.64E-02 | 4 | developmental process | CEBPA\|ADIPOQ\|SLC2A4\|PPARA |
| 8152 | 1.25E-02 | 2.85E-02 | 5 | metabolic process | CEBPA\|ADIPOQ\|SLC2A4\|PPARA\|PNPLA2 |
| 80090 | 1.52E-02 | 3.06E-02 | 4 | regulation of primary metabolic process | CEBPA\|ADIPOQ\|PPARA\|PNPLA2 |
| 50896 | 1.66E-02 | 3.23E-02 | 4 | response to stimulus | CEBPA\|ADIPOQ\|SLC2A4\|PPARA |
| 31323 | 1.84E-02 | 3.52E-02 | 4 | regulation of cellular metabolic process | CEBPA\|ADIPOQ\|PPARA\|PNPLA2 |
| 19222 | 2.20E-02 | 4.02E-02 | 4 | regulation of metabolic process | CEBPA\|ADIPOQ\|PPARA\|PNPLA2 |
| 65007 | 2.69E-02 | 4.63E-02 | 5 | biological regulation | CEBPA\|ADIPOQ\|SLC2A4\|PPARA\|PNPLA2 |
